# Brazilian guidelines on chronic venous disease of the Brazilian Society of Angiology and Vascular Surgery

**DOI:** 10.1590/1677-5449.202300642

**Published:** 2023-11-06

**Authors:** Rodrigo Kikuchi, Claudio Nhuch, Daniel Autran Burlier Drummond, Fabricio Rodrigues Santiago, Felipe Coelho, Fernanda de Oliveira Mauro, Fernando Trés Silveira, Guilherme Peralta Peçanha, Ivanesio Merlo, Jose Marcelo Corassa, Leonardo Stambowsky, Marcondes Figueiredo, Miriam Takayanagi, Ronald Luiz Gomes Flumignan, Solange Seguro Meyge Evangelista, Walter Campos, Edwaldo Edner Joviliano, Walter Junior Boim de Araujo, Julio Cesar Peclat de Oliveira

**Affiliations:** 1 Sociedade Brasileira de Angiologia e Cirurgia Vascular – SBACV, São Paulo, SP, Brasil.; 2 Faculdade de Ciências Médicas Santa Casa de São Paulo – FCMSCSP, São Paulo, SP, Brasil.; 3 Instituto de Excelência Vascular, Londrina, PR, Brasil.; 4 Clínica Vascular, Porto Alegre, RS, Brasil.; 5 Pontifícia Universidade Católica do Rio de Janeiro – PUC-Rio, Departamento de Ciências da Computação, Rio de Janeiro, RJ, Brasil.; 6 Instituto de Doenças Venosas e Linfáticas – IDVL, Goiânia, GO, Brasil.; 7 Pontifícia Universidade Católica do Paraná – PUCPR, Departamento de Cirurgia, Londrina, PR, Brasil.; 8 Universidade Federal de São Paulo – UNIFESP, Departamento de Cirurgia, São Paulo, SP, Brasil.; 9 Clínica Varizemed, Belo Horizonte, MG, Brasil.; 10 Universidade de São Paulo – USP, Faculdade de Medicina, Disciplina de Cirurgia Vascular, São Paulo, SP, Brasil.; 11 Universidade de São Paulo – USP, Faculdade de Medicina – FMRP, Departamento de Cirurgia e Anatomia, São Paulo, SP, Brasil.; 12 Universidade Federal do Paraná – UFPR, Residência em Angiorradiologia e Cirurgia Vascular, Hospital de Clínicas, Curitiba, PR, Brasil.; 13 Universidade Federal do Estado do Rio de Janeiro – UNIRIO, Departamento de Cirurgia Geral e Especializada, Rio de Janeiro, RJ, Brasil.

**Keywords:** venous insufficiency, guideline, varicose veins, venous disease, treatment, insuficiência venosa, diretriz, varizes, doença venosa, tratamento

## Abstract

The Brazilian Society of Angiology and Vascular Surgery has set up a committee to provide new evidence-based recommendations for patient care associated with chronic venous insufficiency. Topics were divided in five groups: 1. Classification, 2. Diagnosis, 3. Conservative or non-invasive treatment, 4. Invasive treatment and 5. Treatment of small vessels. This last series is closely related to the activities of Brazilian angiologists and vascular surgeons, who are heavily involved in the treatment of small superficial veins. These guidelines are intended to assist in clinical decision-making for attending physicians and health managers. The decision to follow a guideline recommendation should be made by the responsible physician on a case-by-case basis taking into account the patient's specific condition, as well as local resources, regulations, laws, and clinical practice recommendations.

## Resumo

A Sociedade Brasileira de Angiologia e de Cirurgia Vascular organizou uma comissão para fornecer novas recomendações baseadas em evidências sobre questões críticas de atendimento ao paciente com insuficiência venosa crônica. São abordados aqui os temas de classificação, diagnóstico, tratamento conservador, tratamento invasivo e tratamento de pequenos vasos. Esta última série está muito relacionada à atividade de angiologistas e cirurgiões vasculares, que possuem forte atuação no tratamento de pequenas veias superficiais. Estas diretrizes destinam-se a auxiliar na tomada de decisões clínicas de médicos assistentes e gestores de saúde. A decisão de seguir uma recomendação de diretriz deve ser feita pelo médico responsável caso a caso, levando em consideração a condição específica do paciente, bem como recursos locais, regulamentos, leis e recomendações de prática clínica.

## INTRODUCTION

Chronic venous disease is a condition affecting the venous system of the lower limbs and may present with various morphological and functional abnormalities.^1^ Its clinical, etiological, anatomical, and physiopathological aspects are described in the CEAP classification.^2,3^ Stages C1 and C2 are the most frequent,^4^ and risk factors include female sex, advanced age, obesity, prolonged standing, positive family history, and parity.^4^ The progression of CVD may be more common in individuals who are overweight and have a history of deep vein thrombosis, and the presence of deep and superficial venous reflux may be associated with new varicose veins.^5,6^

In the most superficial segment, studies show the existence of venous valves in small-diameter veins, and that these valves may also be incompetent.^7^ The incompetence of these micro-valves may play a critical role in the progression of skin changes.^8^ The initial causes of these changes may be inflammatory phenomena^9,10^ which may have both ascending and descending progression.^11^ The disease may also be caused by changes in perforator veins^12^ or post-thrombotic syndromes, the latter with more severe complications.^13^ This communication and the anatomic complexity of the venous system may lead to skin changes, including severe hypoxia with the formation of venous ulcers.^14,15^

The clinical presentations of CVD are varied, and do not necessarily correspond to its clinical severity.^16^ The symptoms are also unspecific, and frequently mistaken for other diseases.^17-19^ CVD is a benign disorder, but which can be correlated with venous thrombosis or bleeding.^20,21^

Scoring systems have been developed in an attempt to monitor treatment outcomes and assess the progression of the disease or other interventions, including the Venous Clinical Severity Score (VCSS), designed to assess changes in patient status after an intervention.^22^ The Villalta scale is used by both the patient and the physician to diagnose and assess the severity of post-thrombotic syndrome.^23,24^ Other tools used to assess CVD include the Aberdeen Varicose Veins Questionnaire (AVVQ),^25^ the Chronic Venous Insufficiency Questionnaire (CIVIQ),^26^ and the Venous Insufficiency Epidemiological and Economic Study - Quality of Life/Symptoms (VEINES-QOL/Sym).^27^

Considering the high prevalence of the disease, with its many nuances in diagnosis and, consequently, in treatment, a national guideline is extremely important to guide practitioners and health system managers.

## METHODS

Vascular surgeons specializing in venous disease and members of the Brazilian Society of Angiology and Vascular Surgery (SBACV) were invited to join the CVD Guidelines Project. All members disclosed their conflicts of interest related to the development of these guidelines. The project received no industry financing. Through biweekly online meetings starting on March 2022, all project members participated in decisions and choices regarding the development of the questions and the organization of this effort.

The group was split into subgroups to optimize the work, namely: 1) classification; 2) diagnosis; 3) conservative or non-invasive treatment; 4) invasive treatment; and 5) treatment of small vessels. Each project member participated in at least two subgroups.

### Selection of recommendations

An initial brainstorming session with all group members was held to select what recommendations would be developed, with each topic then assigned to a subgroup. After this initial stage, the participants of each subgroup were responsible for selecting the most relevant subjects for this guideline.

Search terms were discussed within each subgroup and chosen for queries in the MEDLINE, LILACS, SciELO, and Central databases, in Portuguese, English, and Spanish. Originally, the search period was limited to the period between January 2013 and February 2022, but if search results were not quantitatively or qualitatively sufficient, new queries were performed without date limits.

If required, additional articles were handsearched as well. The article selection and classification process followed an article quality sequence, defined (in descending quality order): systematic reviews, randomized controlled trials, nonrandomized trials, retrospective studies, case series, and expert opinions. When possible, articles were classified for bias risk using Rob 2.0 and Rob cohort (Cochrane, Londres).

### Recommendation criteria

The European Society of Cardiology system was used to grade recommendations according to evidence levels.^28^ These criteria can be found in Tables 1 and 2.

**Table 1:** European Society of Cardiology (ESC) levels of evidence.

**Level of evidence A**	Data derived from multiple randomized clinical trials or meta-analyses.
**Level of evidence B**	Data derived from a single randomized clinical trial or large non-randomized studies.
**Level of evidence C**	Consensus of opinion of the experts and/or small studies, retrospective studies, and registries.

**Table 2:** European Society of Cardiology (ESC) classes of recommendations.

**Class of recommendation**	**Definition**
Class I	Evidence and/or general agreement that a given diagnostic procedure or treatment is beneficial, useful, and effective.
Class II	Conflicting evidence and/or a divergence of opinion about the usefulness or efficacy of the treatment or procedure.
Class IIa	Weight of evidence or opinion is in favor of usefulness or efficacy.
Class IIb	Usefulness or efficacy is less well established by evidence or opinion.
Class III	Evidence or general agreement that the treatment or procedure is not useful or effective and, in some cases, may be harmful.

## CLASSIFICATION

Venous disease is much more common in women than in men. Advanced age and number of pregnancies are important factors for the development of the disease.^29,30^ The CEAP classification, originally established in 1994, was recently revised. Corona phlebectatica was added as C4c due its potential to progress to a venous ulcer.^31,32^

Recommendation 1

- We recommend using the classification of clinical, etiological, anatomical, and physiopathological (CEAP) aspects for all chronic venous insufficiency patients for academic and legal purposes.
  - Level B
  - Class I
  - References:^29,31-34^

Subclass Esi was included to acknowledge intravenous causes, such as post-thrombotic changes and traumatic arteriovenous fistulas. No wall or valve injury was observed in the categorization of extravenous secondary etiologies. The triggers stem from conditions affecting venous hemodynamics.^31^

Clinical presentation is not always indicative of anatomic or hemodynamic severity. A cross-sectional study of 100 patients with varicose veins found no correlation between mean saphenous vein diameter and clinical classification. There was a correlation between advanced age and clinical severity (p = 0.04) and between obesity and greater diameter was measured using ultrasonography.^35^

The primary advantage of CEAP classification is that it uses a single global language. It allows us to state that the physical and functional characteristics were compromised, especially in the more severe forms of CVD,^36^ and that the prevalence of C2 disease is greater in Western Europe and lower in the Middle East and Africa,^34^ and everyone in the scientific community understands what that means.

Recommendation 2

- We recommend using a severity classification score (Venous Clinical Severity Score [VCSS]) for all chronic venous insufficiency patients for academic and legal purposes.
  - Level B
  - Class I
  - References:^37-41^

VCSS correlates well with the CEAP clinical classification and represents a reliable and reproducible tool to document symptom severity in patients with venous insufficiency in the lower limbs.^37^ In an observational cross-sectional study, clinical severity was found to be related to pain, edema, sleep quality, depression, and quality of life for all patients.^38^

VCSS and the Venous Disability Score (VDS) are strongly positively correlated with the Dermatology Quality Life Index (DLQI) and are important tools to assess the severity and impairment of chronic venous insufficiency. This once again stresses the need to develop a classification system for severity, since it is excluded from CEAP.^39,42^

VCSS was created for the purposes of staging and quantifying the natural progression of the disease over time. It enables cost effectiveness comparisons, as well as comparing technical, clinical, and quality of life responses. The suggested updates intend to increase the sensitivity of that assessment tool.^40^

In Brazil, the lack of international consensus on classifications involving CVD has been singled out. An assessment of the scoring system for each revised VCSS criteria separately is provided in a format that could be adopted by the international community.^41^

We suggest using the CEAP classification alongside VCSS. Developing a consensus on an evolutionary assessment of disease progression is also required, as is monitoring treatment outcomes. CIVIQ and AVVQ have been developed specifically for patients with venous disease.

Recommendation 3

- We suggest using a specific system for all patients with telangiectasias or reticular veins.
  - Level C
  - Class IIa
  - References:^43-46^

An open, controlled study attempted to explore the veins of individuals classified as C0S and compare them to C0A (asymptomatic) individuals. Doppler ultrasound identified two different flow patterns: unidirectional and bidirectional. Bidirectional flow was significantly higher (P = 0.05) for C0S compared to C0A patients. This suggests the presence of reflux in non axial veins.^47^

The Bonn Vein Study studied a total of 1350 and 1722 women aged 18 to 79 years old. Leg symptoms (weight, tightness, swelling, pain on standing up or sitting down, pain when walking, muscle cramps, itching, and restless legs) were assessed using a standardized survey; 22.6 percent had varicose veins and 15.8 percent had chronic venous insufficiency (CVI).^46^

Venous disease at several stages has also been found to be associated with cardiovascular death in a cohort study. However, despite different symptoms and clinical manifestations, there is no analysis of CEAP C1 patient scores.^48^

The lesions of CEAP C1 patients clearly have different origins, and saphenous vein reflux is also associated in 40 to 50 percent of patients. This may impact therapeutic decision regarding how these lesions are treated, which would support the need for a separate classification within this clinical class.

CEAP C1-3 patients had less intense reflux scores in Doppler ultrasound than C4-6 patients.^49,50^ This shows the diagnostic utility of Doppler ultrasonography in venous reflux. A literature review including systematic reviews and guidelines concluded that Doppler ultrasonography is the method of choice for CVD diagnosis.^51^

## DIAGNOSTIC TESTS

Vein mapping is essential for surgical planning and should be performed on an individual basis for each patient and treatment technique. Venous reflux is defined as reverse flow time greater than 1 second in the common femoral vein.^51,52^ Doppler ultrasonography enables a topographic and hemodynamic assessment of the deep and superficial systems.

A cohort study of 43 patients surveyed telangiectasias of the lateral thigh by ultrasound. Obese and overweight patients had a higher frequency of incompetent perforator veins and larger reticular veins compared to when compared to those with normal weight (P < 0.05).^53^ The same process was applied to C1 patients to determine whether ultrasound mapping of the saphenous veins is justified. There was a statistically significant tendency that the increased presence of incompetent deep and/or superficial venous incompetence also increases the presence of telangiectasias.^54,55^

An analysis of saphenous vein escape points to reticular veins and small varicosities also found a positive correlation.^56,57^ This shows the importance of using this tool even for early-stage patients.

Recommendation 4

- We recommend using duplex scanning as an early diagnosis tool for all patients with suspected chronic venous insufficiency.
  - Level A
  - Class I
  - References:^49,51,52,58^

Recommendation 5

- We recommend using duplex scanning as an early diagnosis tool for patients with a C1 clinical, etiological, anatomical, and pathophysiological (CEAP) classification.
  - Level B
  - Class IIa
  - References:^53-55,57^

Recommendation 6

- We recommend using abdominal and pelvic venous duplex scanning as an early diagnosis tool for patients with suspected chronic venous insufficiency and suspected suprainguinal stenosis/occlusion.
  - Level B
  - Class IIa
  - References:^59-62^

A Brazilian studied attempted to determine the sonographic criteria for diagnosis of iliac venous outflow obstruction by assessing the correlation of this method with intravascular ultrasound (IVUS) in patients with CVI. The best criteria to detect venous outflow obstruction was a velocity ratio greater than 2.5.^59^ Doppler ultrasound scanning had a positive predictive value of 95.5 percent in detecting more severe stenoses in this segment.^60^

The use of Doppler ultrasonography for iliocaval venous disease has gained popularity in recent years primarily because it is noninvasive. In experienced hands, transabdominal Doppler ultrasonography can consistently show the ovarian veins as well as document their diameter and possible reflux.^60-62^ This enables physicians to avoid more expensive forms of treatment and more time-consuming imaging techniques.

Recommendation 7

- We suggest using other imaging examinations (CT angiography, magnetic resonance angiography and/or venography) in the diagnosis of patients with chronic venous insufficiency and suspected suprainguinal stenosis/occlusion.
  - Level B
  - Class IIb
  - References:^63-68^

Recommendation 8

- We recommend using intravascular ultrasound (IVUS) as an additional investigation method for diagnosis and/or suspected suprainguinal stenosis/occlusion.
  - Level A
  - Class IIa
  - References:^66-71^

Venography does not identify the presence of iliocaval injuries in 19 percent of limbs. The median maximum area stenosis was significantly greater with IVUS than with venography.^63^ Paradoxically, a review found that anteroposterior venography can successfully guide the diagnosis of venous occlusion.^72^

IVUS is consistently superior to venography in detect iliac stenosis.^64-67,69^ Computed tomography (CT) scans can detect iliac vein compression of 50 percent or higher compared to IVUS with 94.09 percent sensitivity and 79.2 specificity. Therefore, though IVUS may be more reliable, CT scans are a possible alternative, especially in cases of stenosis.

Recommendation 9

- We suggest using photoplethysmography as a supplementary diagnosis and therapeutic guidance tool for chronic venous insufficiency patients.
  - Level C
  - Class IIa
  - References:^73,74^

Capillary blood pressure is the primary driving force behind the exchange of fluids between micro-vessels. Subclinical systemic venous congestion, before evident peripheral edema, may directly result in increased peripheral blood pressure. Photoplethysmography (PPG) can be a complementary diagnostic tool for venous insufficiency with functional repercussions.^73,75,76^

Even after treatment, plethysmography found suspected reflux in 71 percent of patients, and precisely those patients had no improvements in quality of life scores. This happened because there was something else wrong: the presence of insufficient perforator veins or residual varicosities in their legs.^74,77^ Therefore, photoplethysmography can be used for both diagnosis and follow-up care.

## CONSERVATIVE OR NON-INVASIVE TREATMENT

In pharmacological treatment, calcium dobesilate was found to be effective in reducing edema in C3-4 patients.^78,79^ For various symptoms, other medications were also efficient, such as red vine leaf extract,^80^ rutosides,^81^ and sulodexide.^82^ All had few adverse effects.

Systematic reviews suggest venoactive drugs probably slightly reduce edema compared to placebos and probably reduce ankle circumference. Gastrointestinal disturbance were the most frequently reported adverse events. The medication of choice in reviews is usually diosmin and hesperidin in micronized fraction.^83-85^

Recommendation 10

- We recommend using venoactive drugs for the symptomatic treatment of chronic venous insufficiency.
  - Level A
  - Class IIa
  - References:^78,79,81-84,86^

Recommendation 11

- We recommend using compression therapy for the symptomatic treatment of chronic venous insufficiency.
  - Level B
  - Class I
  - References:^87-89^

For compression therapy, the use of various grades of compression stockings stands out. The reduction of pain or discomfort and their use in lowering recurrence rates for leg ulcers indicate its use may be positive.^87^

Despite several positive demonstrations, treatment adherence remains a major hurdle, especially when using higher pressures.^88,89^

Physical exercise protocols have shown improvement in range of motion of the tibiotarsal joint and should be considered for treatment of CVI.^90^ Increased muscle strength was found to impact the venous pump, improving its function and the range of motion of the ankle. In addition, pain decreased and quality of life improved after adopting the exercise program.^91-93^ Studies show that physical activity is important to improve venous insufficiency, regardless of intensity.^94,95^

Recommendation 12

- We recommend physical exercise to treat chronic venous insufficiency at any stage.
  - Level B
  - Class I
  - References:^90-97^

Recommendation 13

- We recommend controlling body mass to treat and prevent chronic venous insufficiency at any stage.
  - Level C
  - Class I
  - References:^98-102^

Physical exercise is effective at improving venous reflux, muscle strength, and range of motion of the ankle. Even unsupervised guided exercises can be beneficial.^96-98^

Obesity and reduced mobility, in turn, ran counter to venous return, and obesity contributed to the higher incidence of venous ulcers.^99,103^ Foot venous pressure is significantly higher in obese individuals in all positions. Venous disease is also more severe in obese patients compared to non-obese ones, possibly due to the increase in intra-abdominal pressure. After bariatric surgery in morbidly obese patients, reports of improvements in venous insufficiency are frequent.^100-102,104^

## INVASIVE TREATMENT

### Treatment of saphenous veins

Venous disease is known to be highly prevalent, and its treatment, with the suppression of sites of reflux, has been found to be effective at improving symptoms.^105^ The procedure is also followed by improved quality of life, lower morbidity, and reduced skin ulcers. The total cost for the health system is greater for surgical treatment compared to conservative treatment, but also offers greater health benefits for patients.^106-108^

Recommendation 14

- We recommend invasive treatment with suppression of sites of reflux for patients with symptoms and diagnosis of chronic venous insufficiency.
  - Level A
  - Class I
  - References:^105,107,108^

Invasive treatment is recommended, depending on technical availability, for patients with symptomatic varicose disease. Endovenous laser treatment has been found to be highly effective, with a 92 percent success rate in treating great saphenous vein insufficiency. According to patients, longer wavelengths produce more satisfactory outcomes, and are less painful. Few adverse effects have been observed.^109-112^

Other thermal ablation instruments have been studies, sch as electrocoagulation,^113^ but laser and radiofrequency, with tumescent anaesthesia and without ligation of the saphenofemoral junction (SFJ), have been found to have superior outcomes.^114-117^

Compared to stripping, for instance, thermal ablation has the same long-term success rates, but fewer and less frequent complications in the short term. After 1 year, there were no differences in occlusion rate, and the Aberdeen Varicose Vein Questionaire (AVVQ) 3 months after treatment was similar.^118^

Recommendation 15

- We recommend thermal ablation without SFJ ligation to treat greater saphenous vein (GSV) and small saphenous vein (SSV) insufficiency.
  - Level A
  - Class I
  - References:^108,114,116,118-123^

Recommendation 16

- We recommend stripping to treat GSV and SSV insufficiency.
  - Level A
  - Class IIa
  - References:^108,114,116,118,119,121-124^

In terms of the use of ultrasound guided foam, there are frequent reports of greater recanalization rates compared to other techniques.^119^ The most frequent outcome in systematic reviews and clinical trials is similar long term outcomes for thermal ablation and stripping and inferior outcomes for foam.^120-124^

Specifically for ultrasound guided foam, outcomes are better when tributary veins are treated compared to saphenous or perforator veins.^125-127^ For tributary veins, foam sclerotherapy has durable and impactful results in perceived improvements among patients, despite an expected retreatment rate of 20 percent of limbs within one year.^128^

In the long run, quality scores worsened, requiring therapeutic reintervention, especially in patients with greater vein diameters and distal vein reflux.^129,130^ The primary advantage of using foam is that treatment is easier, does not require anesthesia, and can be repeated (including the possibility of treatment with active ulcers).^131^ Even so, when analyzing long term costs, thermal ablation with local anesthesia are found to have better cost-benefit ratios more frequently. Therefore, that mode of treatment is less effective for patients with large veins and baseline reflux. In a British review, endovenous laser ablation (EVLA) with local anesthesia was considered the most economic strategy overall.^132^

In order to improve sclerotherapy outcomes, there are attempts at using long catheters for foam delivery or combining it with devices utilizing mechanical injury to the vein wall.^133-141^

Though promising, mechanochemical ablation techniques have yet to be found to be at the same level as thermal ablation, with lower occlusion rates. This was observed at 12 to 36-month follow-up.^134-136^

Recommendation 17

- We recommend ultrasound-guided foam sclerotherapy to treat GSV and SSV insufficiency.
  - Level A
  - Class IIb
  - References:^108,116,119-121,126,129,132^

Recommendation 18

- We recommend mechanochemical ablation (MOCA) to treat GSV and SSV insufficiency.
  - Level B
  - Class IIb
  - References:^134-136,139,141^

However, there are positive aspects to these techniques. They are simpler to perform than others, which require a learning curve. Reported complication rates are lower.^138,139^

There are two major schools of thought about the hemodynamics of the superficial venous system. The CHIVA (Cure conservatrice et Hemodynamique de l’Insuffisance Veineuse en Ambulatoire - Conservative Hemodynamic Correction of Venous Insufficiency) procedure is a surgical intervention to repair abnormal hemodynamic vessels. ASVAL (Ablation Sélective des Varices Sous Anesthésie Locale — Selective Varicose Vein Ablation under Local Anesthesia) proposes the multifocal ascending theory, and the pressure of the blood column, combined with the weakness of the vein wall, creates a varicose reservoir.^142-148^

Recommendation 19

- We suggest saphenous vein preservation surgery to treat chronic venous insufficiency.
  - Level C
  - Class IIb
  - References:^142,143,145^

When comparing CHIVA, conventional surgery and EVLA, CHIVA and EVLA have better aesthetic outcomes and are less painful. Despite the benefits suggested by CHIVA, it requires a longer learning curve and greater surgeon expertise than venous hemodynamics.^142,145^

The CHIVA technique is based on the concept of hemodynamics, treating venous shunts with ligation of escape points and preservation of saphenous veins. The results also indicate reduced diameter, with an acceptable recurrence rate.^143-145^

### Treatment of perforator veins

Foam sclerotherapy can be recommended as a first-line treatment for perforator veins since it is minimally painful and less expensive than other forms of treatment. Unlike outcomes for saphenous veins, even thermal ablation can have disappointing success rates.^149,150^

Recommendation 20

- We suggest the use of thermal ablation to treat perforator veins, when indicated.
  - Level C
  - Class IIa
  - References:^150,151^

Recommendation 21

- We suggest the use of foam sclerotherapy to treat perforator veins, when indicated.
  - Level C
  - Class IIa
  - References:^152,153, 206, 207^

Occlusion rates for perforator veins range from 30 to 70 percent, with improvements when treatment is repeated.^151,154-156^ The need to treat perforator veins is still controversial. Laser or radiofrequency (RF) thermal ablation has a long learning curve, and technical skill can be an important hurdle. For many, however, foam is the first choice, because it is less invasive and more easily performed.^207^ Occlusion rates are slightly worse, but ease of reintervention makes it the most frequently used technique.^154-156, 206, 207^

### Treatment of tributary veins

The outpatient phlebectomy procedure was first described many years ago and has changed little over time, with widely known results. However, other modes of treatment have garnered interest in the area, such as foam sclerotherapy or even endovenous laser.^157-174^

Recommendation 22

- We recommend phlebectomy to treat tributary veins.
  - Level B
  - Class I
  - References:^157,158,161^

Recommendation 23

- We suggest endovenous laser as an alternative to treat tributary veins.
  - Level C
  - Class IIb
  - References:^165,167^

Recommendation 24

- We recommend foam sclerotherapy to treat tributary veins.
  - Level B
  - Class IIa
  - References:^158,163,173^

The outpatient phlebectomy procedure was first described many years ago and has changed little over time. It reigned for many years as the standard treatment technique for tributary veins. The procedure is performed safely and effectively in outpatient settings.^158,159^

Both 1-year and 2-year recurrence rates are small for phlebectomy, though there are complications inherent to the surgical procedure. These situations are not present in foam sclerotherapy treatment, for instance.^157,161^

The use of sclerotherapy to treat tributary veins is well-known and has been used for many years in phlebology. It can use a wide range of concentrations, both in liquid and foam mode, and is effective.^168,170,171^ However, its most common adverse event, superficial thrombophlebitis, is extremely unpleasant, and increases the risk of pigmentation.

For larger extents and a greater number of tributary veins, foam volume may be an obstacle.^172,173^ Foam sclerotherapy and phlebectomy, when used for tributary veins combined with endothermal approaches for saphenous veins, has high rates of success, preferably performed as a single procedure.^157,162,163,169,170^

There is little evidence of the benefits of using endovenous laser for tributary veins. The longer technique, higher costs, and rate of post-procedure hardening make it unpopular among experts. In addition, there are lingering questions about the power used and the proper indications for that mode of treatment.^164,165,167^

Recommendation 25

- We suggest the use of intra-procedural Doppler ultrasound during invasive vein treatment.
  - Level C
  - Class I
  - References: ^176,consensus^

The chronic nature of the disease makes it difficult to assess efficacy, but follow-up of high patient-satisfaction cases make it an interesting alternative. With the development of new endovenous techniques, the need for real-time treatment monitoring has emerged. The benefits of ultrasound-guided puncture is clear, with an 82 percent decrease in failure rates.^174-176^ In fact, procedures are already described as ultrasound-guided, which makes them inherent to the performance of the procedure. In other words, the procedure would not be possible without ultrasonography.

## TREATMENT OF SMALL VESSELS

Sclerotherapy has a wide range of applications in the treatment of cutaneous telangiectasia, superficial venous insufficiency, pelvic venous reflux, and venous malformations. Strokes, the most feared complication from sclerotherapy, are fortunately rare. Migraines and visual disturbances, however, are reported more frequently.^177^

Recommendation 26

- We recommend liquid chemical sclerotherapy to treat chronic venous disease C1.
  - Level A
  - Class IIa
  - References:^178-183^

Recommendation 27

- We recommend the use of transcutaneous laser treatment for chronic venous disease C1, especially for telangiectasias.
  - Level B
  - Class IIa
  - References:^182-184^

Liquid sclerotherapy is recommended for smaller reticular veins, venulectasia, and telangiectasia. Setting realistic expectations for patients relieves anxiety and enables a positive treatment experience.^178^ Few post-sclerotherapy or thrombophlebitis complications are observed in ulcers, while hyperpigmentation is the most frequent minor adverse event.^179,185^

Though there is no clear evidence of superiority among sclerosants, superior results tend to be observed with the use of a detergent (such as polidocanol) compared to the use of a hypertonic agent (such as 75% glucose) alone.^180,181^

The use of transcutaneous laser treatment has grown, especially for telangiectasias. However, results vary widely, depending on parameter used, wavelength, and agent compared.^182-184,186-190^

The 1064 nm Nd:YAG long pulse laser is the most widely used to treat leg veins, but still requires further studies and assessment. One of the primary advantages of transcutaneous laser treatment is the possibility to treat lesions for which sclerotherapy is not a viable choice.^189^

Recommendation 28

- We suggest radiofrequency transcutaneous treatment for C1 chronic venous disease, especially for telangiectasias.
  - Level C
  - Class IIa
  - References:^191^

Recommendation 29

- We suggest the use of laser associated with chemical sclerotherapy to treat chronic venous disease C1.
  - Level C
  - Class IIb
  - References:^192-194^

Radiofrequency treatment can also be used to treat small vessels. Outcomes for standalone treatment and in combination seem promising, but still require more robust studies before it can be indicated and become better known.^184,190,191^

The combination of laser and liquid or foam sclerotherapy has been widely used and was highly recommended until recently. However, studies of the associated procedures are limited to a few case series, and though most have very promising results, others have had disastrous complications.^192-194^

Recommendation 30

- We suggest compression therapy after treatment of small vessels with sclerotherapy, laser, or radiofrequency.
  - Level B
  - Class IIb
  - References:^195-199^

Recommendation 31

- We suggest against the routine use of topical medications after all modes of treatment for venous disease C1.
  - Level C
  - Class III
  - References:^200-202^

The use of compression therapy with compression stockings is controversial. Though previous studies have found the possibility of better outcomes, recent randomized studies did not corroborate those findings.^196-199^

Therefore, the use of compression therapy after treatment of small vessels is still debatable. Their actual benefit is hard to explain when we know the pressure required for vessel collapse is very high. Other authors state that costs are costs and benefits uncertain.^198,199^

Routine use of topical ointments and medications after treatment of small vessels is unsupported in the scientific literature. There is no evidence that the use of substances such as corticosteroids, arnica or bromelain provide any benefits. Some may actually be harmful.^200-204^ There are reports of the use of topical substances in the attempt to reverse some complications, such as matting, but still no significant evidences.^203,204^

After treatment, the recommendation is that patients use sunscreen and moisturizers, but, even so, there is no evidence of improved results or lower complications rates.^205^

## FINAL CONSIDERATIONS

The goal of this project was to provide guidelines for professionals and for the population in general when faced with a wide varied of situations involving an extremely common illness. At no point did the recommendations provided here intend to become absolute rules for medical practice. Rather, their goal was to help attending physicians make the best decision for their patients.

The development of these recommendations followed an extensive review of scientific publications, combined with expert opinions when the evidence was scarce or conflicting.

There are still gaps in scientific knowledge, and further publications in this area are needed. Therefore, these recommendations should be reviewed and revised periodically in light of new evidence.
